# Characterization of the Tumor Microenvironment in Osteosarcoma Identifies Prognostic- and Immunotherapy-Relevant Gene Signatures

**DOI:** 10.1155/2022/6568278

**Published:** 2022-08-27

**Authors:** Jianye Tan, Xuhui Feng, Hangxing Wu, Bingsheng Yang, Meiling Shi, Chao Xie, Zexin Su, Lin Li, Mengliang Luo, Zhijie Zuo, Shuang Zhu, Jiancheng Yang, Lijun Lin

**Affiliations:** ^1^Department of Orthopedics, Zhujiang Hospital, Southern Medical University, Guangzhou, Guangdong, China; ^2^Department of Orthopaedic, Huizhou First Hospital, Guangdong Medical University, Huizhou, 516003 Guangdong, China; ^3^Division of Orthopaedics and Traumatology, Department of Orthopaedics, Nanfang Hospital, Southern Medical University, Guangzhou, China; ^4^Department of Rheumatology and Clinical Immunology, Jiangxi Provincial People's Hospital Affiliated to Nanchang University, Nanchang, China; ^5^Department of Musculoskeletal Oncology, The First Affiliated Hospital of Sun Yat-sen University, Guangzhou, Guangdong, China

## Abstract

The osteosarcoma (OS) microenvironment is composed of tumor cells, immune cells, and stromal tissue and is emerging as a pivotal player in OS development and progression. Thus, microenvironment-targeted strategies are urgently needed to improve OS treatment outcomes. Using principal component analysis (PCA), we systematically examined the tumor microenvironment (TME) and immune cell infiltration of 88 OS cases and constructed a TME scoring system based on the TMEscore high and TMEscore low phenotypes. Our analysis revealed that TMEscore high correlates with longer survival in OS patients, elevated immune cell infiltration, increased immune checkpoints, and increased sensitivity to chemotherapy. TMEscore low strongly correlated with immune exclusion. These observations were externally validated using a GEO dataset (GSE21257) from 53 OS patients. Our laboratory data also proved our findings. This finding enhances our understanding of the immunological landscape in OS and may uncover novel targeted therapeutic strategies.

## 1. Introduction

Osteosarcoma (OS) is the most common primary malignancy of the bone [1–3]. OS is characterized by a relatively high degree of malignancy with a predilection for children and adolescents. Although its morbidity rate is low, metastatic spread and chemotherapy resistance are the main cause of OS-associated mortality. Although neoadjuvant chemotherapy in combination with surgery has improved the survival rate of OS, and its 5-year survival rate remains low.

Due to their low immunogenicity, tumor cells often escape the host's immune system [4, 5]. Tumor immunogenicity is mediated by tumor-associated immune cells and TME. The possible explanation of antitumor immune response is that tumor cells evade TME surveillance, leading to uncontrollable disease in the subsequent stage [6]. In other words, a tumor exploits immune checkpoint pathways to regulate tumor-reactive T cells, creating a TME that is conducive for cancer growth and metastasis [7, 8].

The TME is characterized by a complex relationship between TME-resident cell types and tumor cells [9–11]. Numerous studies have investigated the relationship between the TME and impact of patient prognosis [12–14]. Thus, in order to improve OS survival rates, a better understanding of its immune microenvironment is needed. This would elucidate novel ways of preventing tumor recurrence and metastasis and promoting tumor cell apoptosis and differentiation, thereby improving patient survival and quality of life.

Recent studies indicate that tumor cells also can contribute to resistance to chemotherapy by modifying the TME. Although immunotherapy has been developed for treating OS, it is difficult to predict which patients it is likely to benefit [15]. Considering that the TME is highly heterogeneous, it is necessary to characterize the TME in individual tumor.

However, few studies have investigated the TME in OS. Here, we sought to characterize TME subtypes in OS that exhibit disparate biological behaviors. We also investigated accuracy and rationality classification of TME subtypes, OS responses to therapy, and clinical outcomes.

## 2. Materials and Methods

### 2.1. Raw Data

The RNAseq data analyzed in this study was from The Cancer Genome Atlas OS cohort (TCGA-OS) obtained from 88 OS samples. Dataset GSE21257 (*n* = 53) was downloaded from GEO and used for external validation. SVA, an R package, was used to merge raw data from both datasets and to eliminate batch-to-batch differences [16].

### 2.2. Evaluation of the Immune Score

We used the ESTIMATE algorithm, which quantifies the proportion of TME in OS samples from cell transcripts [17]. Based on median values, all samples were categorized into high score and low score groups.

### 2.3. Single-Sample Gene Set Enrichment Analysis (ssGSEA)

Twenty-nine immune datasets, including category of immune cell, immune-related functions, and immune-related pathways, were subjected to ssGSEA in order to determine population-specific immune infiltration [18]. Genetic characterization of immune cell population expression in individual cancer samples was obtained using ssGSEA. Based on ssGSEA results, OS cases in TCGA dataset were classified into the high or low immune cell infiltration groups using “hclust.”

### 2.4. Identification of Immune-Related Genes in the Low and High Immune Score Groups

TCGA transcriptomic data from 88 OS patients was divided into the top and bottom parts (high vs. low immune score group and high vs. low immune cell infiltration group) based on their immune category. Then, the identification of immune-related genes (IRGs) was achieved by using LIMMA analysis [19] to compare standardized expression data between high and low immune groups. ∣logFC | >1 and adjusted *p* < 0.05 were set as cutoffs for identifying IRGs.

### 2.5. Inference of Infiltrating Cells in the TME

We used CIBERSORT and the LM22 gene signatures to determine the proportion of immune cells in OS samples, which allows for high sensitivity and specific discrimination of 22 human immune phenotypes, including B cells, natural killer cells, macrophages, T cells, myeloid subsets, and dendritic cells (DCs). CIBERSORT is a deconvolution algorithm based on a set of reference gene expression values (signatures of 547 genes), which are considered to be the minimum representation of each cell type. On this basis, the proportion of cell types in tumor sample data with mixed cell types was inferred based on support vector regression [20].

### 2.6. Identify Genes Related to Immune Cell Infiltration

Spearman correlation analysis was used to identify genes highly correlated with immune cell infiltration (∣*R* | >0.3, *p* < 0.05). Univariate Cox regression analysis was used to assess the impact of IRGs on patient survival.

### 2.7. Dimension Reduction and Generation of TME Gene Signatures

To construct TME metagenes, we first used LASSO (least absolute shrinkage and selection operator) analysis using the “glmnet” package on R for dimension reduction so as to minimize noise from redundant genes [21]. Next, each member of the immune cell-related genes was standardized across all samples in the OS datasets (TCGA OS and GSE21257). The hub genes were then analyzed using unsupervised clustering (*K*-means), and the patients were divided into several subgroups for further analysis. The cluster profiler package on R was then used to annotate gene clusters [22]. Gene clustering was determined by consensus clustering algorithm. PCA analysis was then performed on each patient to determine the TMEscore of each patient:

(1) $$\begin{array}{c} \text{TMEscore}={\text{PCi}}_{\text{A}}-{\text{PCi}}_{\text{B}}. \end{array}$$

### 2.8. GSVA

Differences in biological processes between the low- and high-TMEscore groups were analyzed using the “GSVA” package on R [23]. GSVA is a nonparametric and unsupervised approach to evaluating pathway changes or biological processes by expressing matrix samples. The “C2. Cp.kegg. V7.2. Symbol” gene set is used as the reference gene set from the molecular signature database (https://www.gsea-msigdb.org/gsea/msigdb). For each statistically significant enrichment pathway, adjusted *p* < 0.05 was considered statistically significant.

### 2.9. Exploration of the Significance of TMEscore in Predicting Response to Chemotherapy and Targeted Therapy

To assess TMEscore's capacity to predict clinical response to OS treatment, we calculated the IC50 of common chemotherapeutic drugs and targeted agents in tumor clinical treatment, including axitinib, AZD6244, CI.1040, cisplatin, and JNK inhibitor VIII. The difference in IC50 between the high- and low-TMEscore groups was determined by Wilcoxon signed-rank test and the R packages pRRophetic and ggplot2 [24, 25] used to visualize the results.

### 2.10. Prediction of Immunotherapy Response

Next, we evaluated the correlation between OS response to immunotherapy and TMEscore. The tumor immune dysfunction and exclusion (TIDE) scoring system [26] (http://tide.dfci.harvard.edu/) was used to evaluate responses to immunotherapy in the TMEscore subgroups. The higher the immune exclusion score, the worse the tumor response to immunotherapy and the worse the prognosis. Kaplan-Meier (KM) analysis was used to determine overall survival in patients after stratification by TME score. Differences were considered statistically significant when two-tailed *p* < 0.05. Violin plot analysis using the ggstatsplot package on R was used to study the relationship between the subgroup and the expression level of immune checkpoint genes, including CD274, PDCD1LG2, CD27, CTLA-4, LAIR1, and TIGIT [27–30].

### 2.11. Human OS Tissue Collection

In the present study, the consent forms must be signed before each patient is included in the present study and all aspects of the study were approved by the Ethics Committees of Zhujiang Hospital, Southern Medical University (no. 2018-GJGBWK-002). We collected the tissues of 36 patients with OS from Zhujiang Hospital.

### 2.12. RNA Extraction and Gene Expression Measurements

According to the manufacturer's instructions, the RNAiso Plus Reagent Kit (Accurate Biotechnology (Hunan) Co., Ltd., China) was used to extract the total RNA in the tissues of OS patients. After quantification of RNA, cDNA was synthesized using PrimeScript RT Reagent Kit (TaKaRa, Japan). Subsequently, RT-PCR was performed using the SYBR Premix Ex Taq II (TaKaRa, Japan). The primers were as follows: GAPDH forward: 5′-GGAGCGAGATCCCTCCAAAAT-3′ and GAPDH reverse: 5′-GGCTGTTGTCATACTTCTCATGG-3′; EVI2B forward: 5′-AAGCAGTCACAGCCTACCTTA-3′ and EVI2B reverse: 5′-TGAATTGTGTTGGTTGACCCAAA-3′; and CD4 forward: 5′-TGCCTCAGTATGCTGGCTCT-3′ and CD4 reverse: 5′-GAGACCTTTGCCTCCTTGTTC-3′.

### 2.13. Cells and Cell Culture

Human OS cells (143B and MNNG) were obtained from American Type Culture Collection (ATCC, Manassas, US) and cultured in DMEM (Invitrogen, US) supplemented with 10% FBS and 1% penicillin/streptavidin (Gibco), at 37°C [31].

### 2.14. OS Cell Transfection

The cells were cultured in a 6-well culture plate and washed with PBS before lentivirus transduction; then, the overexpressed lentivirus carrying EVI2B was cultured with composite transfer solution for 24 hours, and then, the cells were cultured in puromycin (2 *μ*g/mL, Invitrogen, USA) selective medium to screen stably transfected cells.

### 2.15. Colony Formation Assay

800 transfected cells were seeded onto 6-well plates and cultured at 37°C for 7 days. The cells were then treated first with 4% paraformaldehyde for 25 minutes, followed by 0.1% crystal violet for 15 minutes. Finally count the number of colonies with the help of a scanner. The experiment was done in 3 independent replicates.

### 2.16. Cell Migration and Invasion Assays

600 *μ*L DMEM of 10% fetal bovine serum was added into the lower lumen of Transwell. 200 *μ*L DMEM containing 5 × 10^4^ cells was inoculated into the upper lumen of Transwell. They were then cultured at 37°C for 24 h before removing the cells on the upper surfaces of the filter using cotton swabs. Cells that migrate to the lower surface of the chamber were then fixed with 4% paraformaldehyde for 20 min and stained with 0.1% crystal violet for 15 min, and finally, the chambers were moved to the microscope for recording. The experiment was done in 3 independent replicates.

### 2.17. Apoptosis Assay

Annexin V-APC/PI apoptosis detection kit was used for apoptosis analysis (BestBio, Shanghai, China) and flow cytometry. OS cells that had been transfected for 24 h were cultured in serum-free media for 24 h and 1 × 10^5^ cells stained using Annexin V/APC and PI for 10 min in the dark. The percentage of apoptotic cells was then detected using CytExpert and analyzed using FlowJo.

### 2.18. Animal Experiments

Nude mice (male, 4 weeks old) were used as *in vivo* tumor models. 100 *μ*L of cells transfected with NC or the overexpression of EVI2B (OE-EVI2B) vector were then injected subcutaneously into nude mice at 1 × 10^7^ 143B cells/mL. After 50 days, all mice were euthanized via CO_2_ inhalation. Mouse experiments were approved by the animal experiment ethics committee of Zhujiang Hospital.

### 2.19. Statistical Analysis

The experimental results were compared between the two groups by two independent sample *t*-test. Data were presented as mean ± SD. The difference in survival between the low- and high-TMEscore groups was assessed using the long-rank test. Statistical analyses were performed in R version 4.1.1 (https://www.r-project.org/) or SPSS version 25.0. *p* < 0.05 indicated statistical significance.

## 3. Results

### 3.1. Immune-Related Signaling Pathways Are Markedly Activated in OS Patients

**Figure 1:**
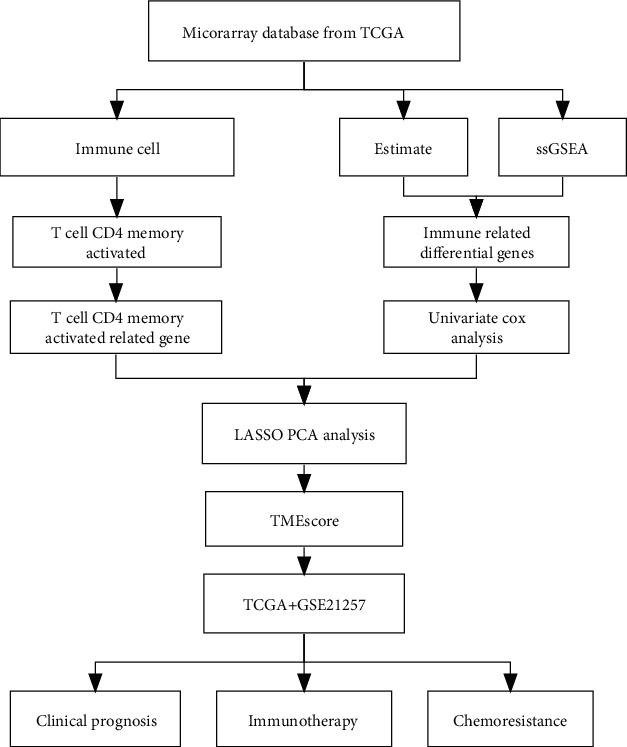
Flow diagram of the study design.

**Figure 2:**
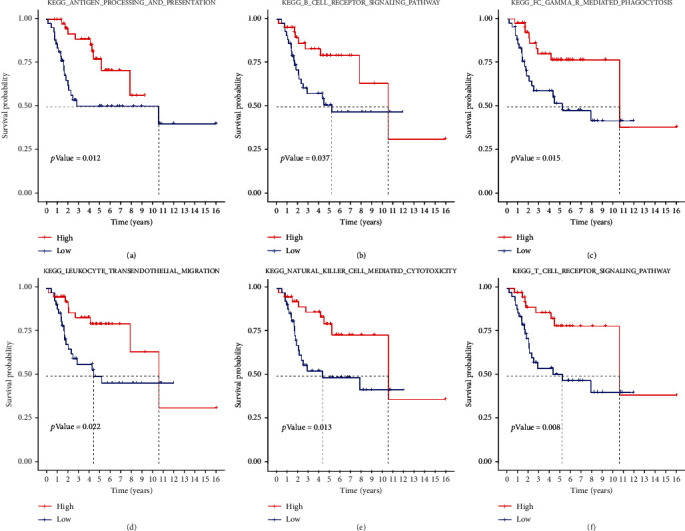
Immune signaling pathways associated with survival in OS patients.

The flow chart of this study is shown in Figure 1. To elucidate the biological behavior among these different biological molecular patterns, we performed GSVA analysis of hallmark gene sets in OS patients and identified 5 significant immune-related pathways (KEGG_Antigen_Processing_And_Presentation, KEGG_B_Cell_Receptor_Signaling_Pathway, KEGG_FC_Gamma_R_Mediated_Phagocytosis, KEGG_Leukocyte_Transendothelial_Migration, KEGG_Natural_Killer_Cell_Mediated_Cytotoxicity, and KEGG_T_Cell_Receptor_Signaling_Pathway) related to overall survival of OS patients (Figure 2). We found that immune-related signaling pathways can affect the survival of OS patients, so we carried out a series of studies to explore the relationship between immunity and OS patients.

### 3.2. Two Different TME Exist in OS

**Figure 3:**
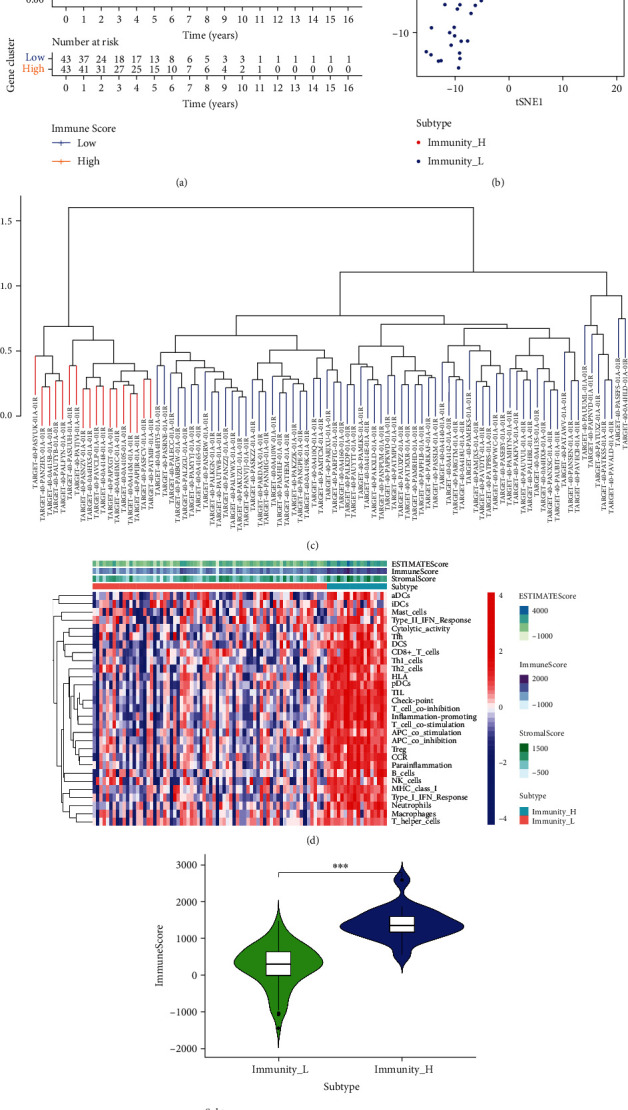
Elucidation of the immune cell infiltration landscape in OS. (a) ESTIMATE was used to evaluate survival in OS patients belonging to the high- and low-TME subgroups. (b) TSNE confirmed the rationality of ssGSEA grouping. (c) Based on the ssGSEA results, OS patients were divided into the immunity_ H and immunity_ L groups. (d) Landscape of the immune characteristics and TME in TCGA-OS cohort. (e) Correlation analysis between the ESTIMATE algorithm method and ssGSEA grouping.

Considering the important immune role played in OS patients, we applied the ESTIMATE algorithm to assess TME and are aware of understanding the potential relationships between prognostic-related TME; finding a high immune score group was more likely to achieve a better prognosis than the low immune score group (Figure 3(a)). Since immunocyte are the components of TME, we began to consider whether there was a potential connection between TME infiltrate immune cells and the TME.

Next, we used the ssGSEA algorithm to quantify tumor immune cell infiltration and its impact on OS patients. Using unsupervised hierarchical clustering, the OS samples were divided into the high (*n* = 18) and low immune cell infiltration groups (*n* = 70) based on the level of immune infiltration (Figures 3(b) and 3(c)). Data from both methods were relatively consistent, indicating dense reciprocal connections between immunocyte and TME. The group with more immune cell infiltration had higher immune microenvironment score (Figures 3(d) and 3(e)).

### 3.3. Identification of Immune Genes Associated with Memory CD4+ T Cells

**Figure 4:**
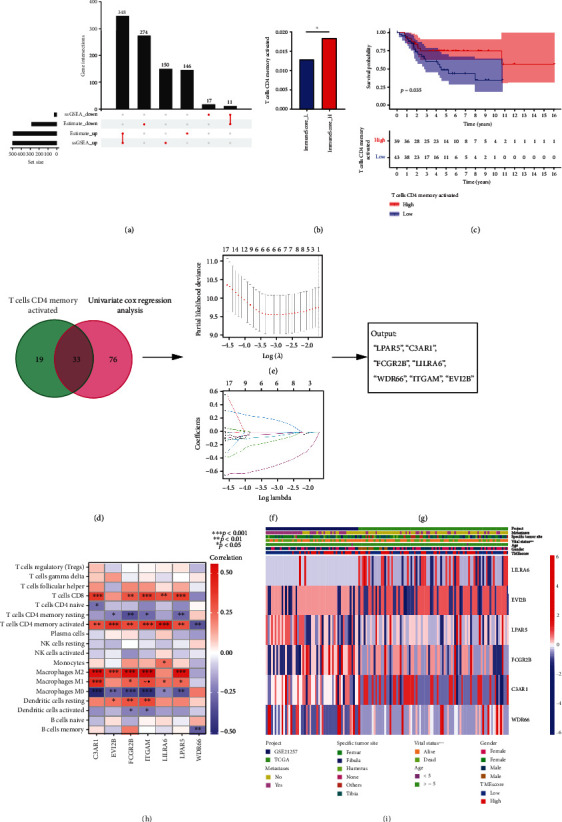
Construction of the TMEscore gene signature. (a) The UpSet plot compared high- and low-expression groups as determined using ESTIMATE and ssGSEA and identified 359 interaction genes. (b) CD4+ TMA was differentially expressed in the high versus low immune score groups. (c) CD4+ TMA affects prognosis. (d–g) Venn plots showing the number of interaction genes that are closely related to CD4+ TMA and univariate Cox regression analysis. These genes were subjected to LASSO analysis. *X*-axis: -log (lambda), *Y*-axis: LASSO coefficients. LASSO analysis identified 7 genes which were subjected to follow-up analyses. (h) Analysis of correlation between the 7 genes and immune cells. (i) Heatmap visualization of the relationship between the 7 genes and clinical features.

Two algorithms (ESTIMATE and ssGSEA) for evaluating tumor immune environment all divided TCGA-OS into high- and low-expression groups. Firstly, we find the difference genes (DEGs) between high- and low-expression groups of each algorithm. Then we find the common genes between the two algorithms. Finally, we focused on these intersection DEGs in subsequent analysis (Figure 4(a)).

We also used CIBERSORT to validate the above groups and found that the high immune cell infiltration group had higher levels of immune cells. Notably, CD4 T cells, memory activated (CD4+ TMA) significantly correlated with prognosis (Figure 4(c)). We also observed significant differences between CD4+ TMA levels in the high and low immune score groups (Figure 4(b)).

Following this, Spearman correlation analysis was used to identify genes closely related to DEGs and CD4+ TMA infiltration, and it is considered that more attention should be paid (Figure 4(d)).

### 3.4. Identification of Immune Genes Associated with OS Subtype-Specific Immunity

The prognosis of OS patients is the most important issue for clinicians and patients; catering to the clinical need is the starting point of the research. To identify genes that may affect survival of OS patients, we employed univariate Cox regression analysis and identified 109 genes that were associated with patient outcomes (Figure 4(d)).

Venn diagram analysis revealed the number of prognostic genes and their intersection with strong CD4+ TMA-correlated gene modules. These genes were further input into LASSO analysis. After LASSO analysis, C3AR1, EVI2B, FCGR2B, LILRA6, LPAR5, ITGAM, and WDR66 were selected for subsequent analysis (Figures 4(e)–4(g)). Analysis of gene function implicated these genes in immune cell infiltration and the clinical characteristics of OS patients (Figures 4(h) and 4(i)). C3AR1, EVI2B, FCGR2B, LILRA6, LPAR5, and ITGAM were significantly positively correlated with antitumor CD8+ T cells.

### 3.5. Development of the TME Scoring System for OS

**Figure 5:**
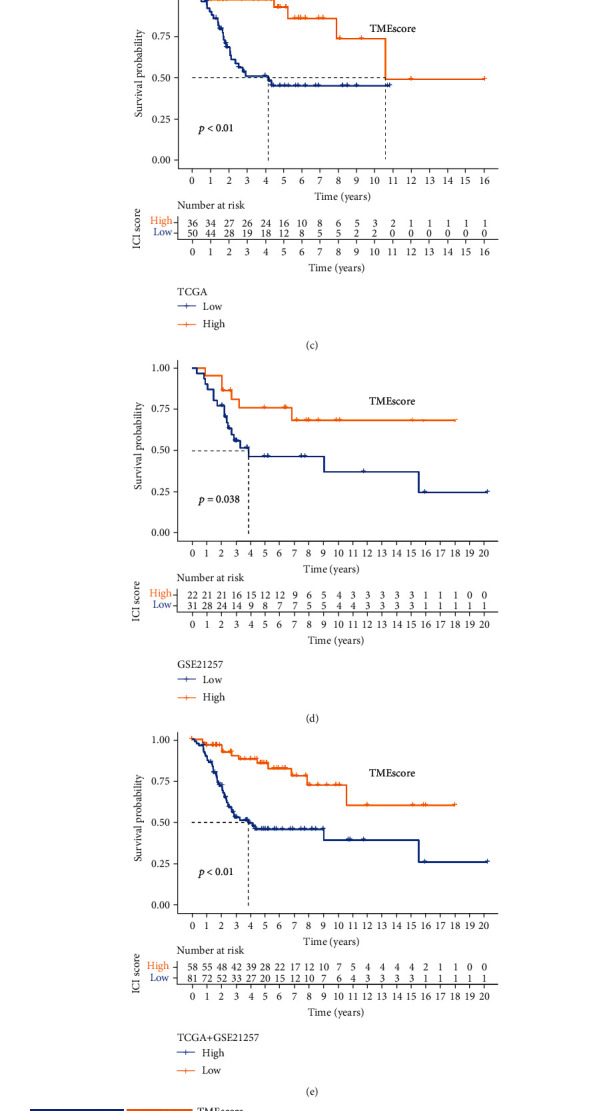
Construction and validation of TMEscore for predicting clinical outcome. (a) Heatmap of consensus matrix (consensus matrix *K* = 2). Unsupervised clustering based on 7 genes divided the samples into two categories. (b) Alluvial representation of the relationship between TME subtypes, TMEscore subgroups, and clinical outcomes. (c) KM analysis of high- and low-TMEscore subgroups in TCGA cohort (d) and external validation cohort (GSE21257). (e) KM analysis of high- and low-TMEscore subgroups in all OS cohorts (TCGA and GSE21257). (f) GSVA enrichment analysis showed the activation status of signaling pathways in various TMEscore subgroups. Heatmap visualization of these biological processes. Red indicates activated pathways. Blue indicates suppressed pathways.

To identify the underlying biological characteristics, based on 7-gene expression profiles, we used the PCA algorithm to recluster the 7 genes into genomic subtypes (gene clusters A and B, Figure 5(a)). TME cluster A contains EVI2B and LILRA6, and TME cluster B contains C3AR1, FCGR2B, ITGAM, LPAR5, and WDR66. We then computed the TMEscore of each OS sample using the algorithm that “TMEscore A obtained from TME cluster A” was subtracted from “TMEscore B obtained from TME cluster B” (Figure 5(b)). The OS samples were divided into high TMEscore and low TMEscore using the survival package and an optimal cutoff of 0.6643204 (Figure 5(c)).

Evaluation of the survival rate of the two immune subtypes revealed that high-TMEscore patients had the best prognosis. To validate the robustness of the criteria for TME grouping, we performed the same statistical analyses in another OS cohorts (GSE21257, Figures 5(d) and 5(e)) and observed that TMEscore retained prognostic significance.

Next, we used GSVA enrichment analysis to examine biological processes and KEGG pathways associated with the TMEscore. This analysis revealed that both OS cohorts (TCGA and GSE21257) were enriched for various biological processes related to immune and inflammatory responses, folic acid metabolism, and apoptosis. In addition, differences were found between immune subtypes. Various signaling pathways, including JAK/STAT, TOLL LIKE RECEPTOR, and NOD LIKE RECEPTOR signaling were also enriched in the high-TMEscore group (Figure 5(f)).

**Figure 6:**
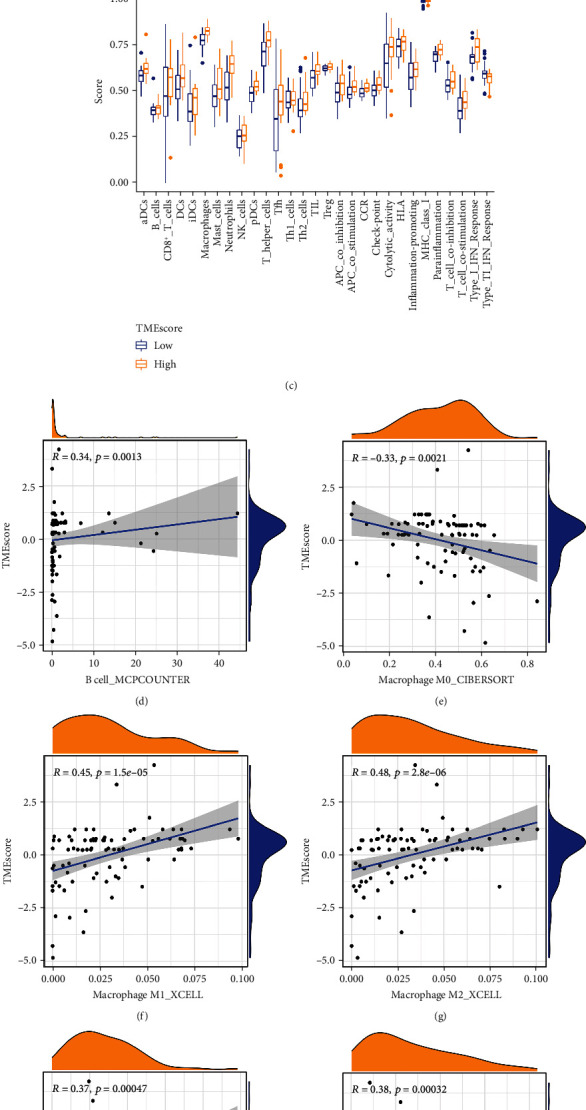
Immune-related characteristics of the TMEscore. (a) Heatmap of immune responses based on TIMER, CIBERSORT, CIBERSORT-ABS, QUANTISEQ, XCELL, EPIC, and MCPCOUNTER analyses of the high- and low-TMEscore groups. (b, c) Expression of immune cells and immune-related pathways in the two clinical cohorts. (d–o) Analysis of correlation between TMEscore and immune cell infiltration.

Next, we used ssGSEA to identify immune cells and immune-related pathways in the subtype of immune. This analysis showed that in the high score subtype of TMEscore, nearly all immune cells and immune-related pathways were upregulated (Figures 6(b) and 6(c)). Multiple deconvolution approaches (TIMER, CIBERSORT, CIBERSORT-ABS, QUANTISEQ, XCELL, EPIC, and MCPCOUNTER (20-24)) were used to estimate the abundance of immune cell infiltration in the OS cohorts (TCGA and GSE21257, Figure 6(a)). To examine the heterogeneity of immune responses in different subtypes, we analyzed correlation between TMEscore and immune cell infiltration and observed that TMEscore level positively correlated with macrophage, monocyte, myeloid dendritic cells, and B cells but negatively correlated with macrophage M0 (Figures 6(d)–6(o)).

### 3.6. Predictive Value of TMEscore as a Biomarker for Therapeutic Effect

**Figure 7:**
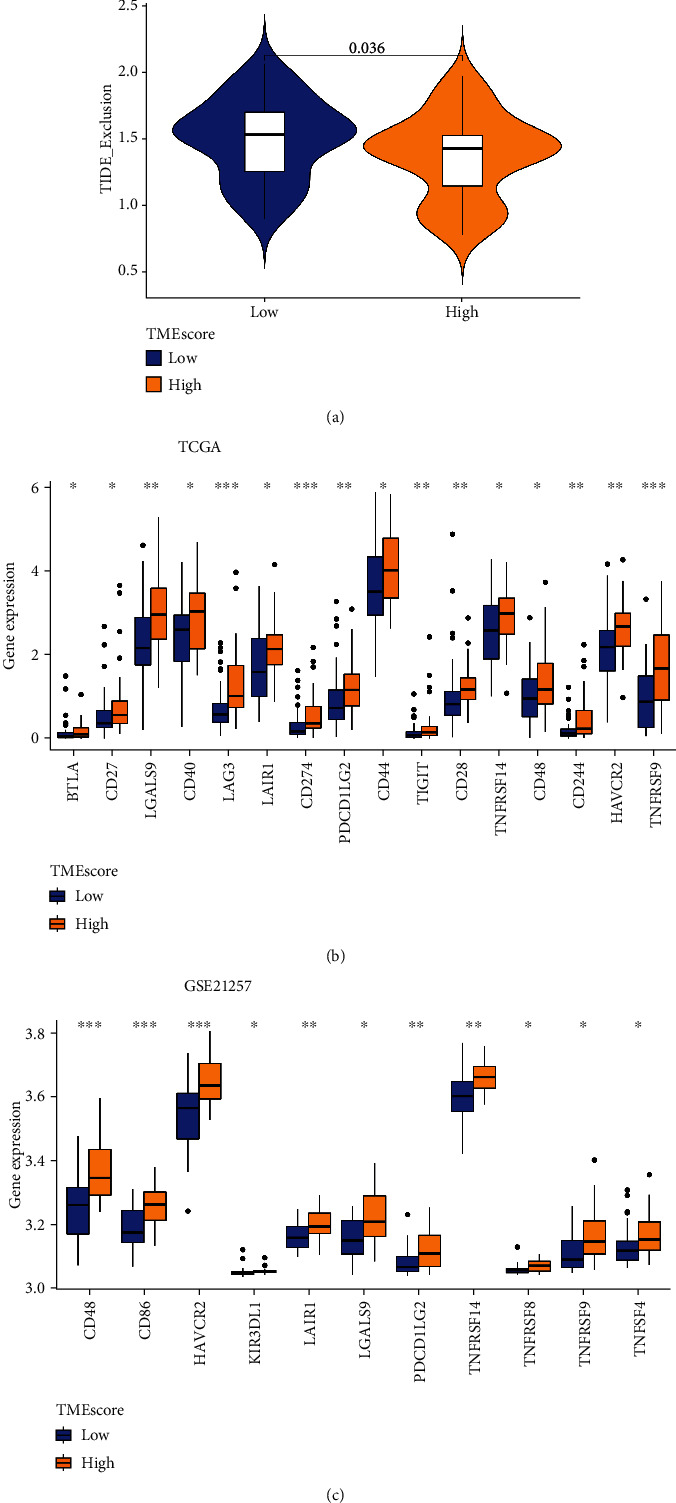
Potential immune escape mechanisms associated with the TMEscore. (a) Immune exclusion score in high and low TMEscore. (b) Expression of immune checkpoints was higher in the high-TMEscore group relative to the low-TMEscore group in TCGA cohort. (c) Immune checkpoint expression was validated using dataset GSE21257.

To obtain further proof that TMEscore is an effective predictor of immunotherapy success, we used TIDE to predict the response of tumor immune exclusion. We found that the lower the score of TMEscore, the more prone to immune exclusion. This analysis revealed the biomarker potential for TMEscore in immunotherapy (Figure 7(a)).

Using TMEscore as a predictor of immunotherapy, we observed differential expression of various immune checkpoints in the high- vs low-TMEscore subgroups, with most immune checkpoint- and immune activity-related genes, including CD48, HAVCR2, LAIR1, LGALS9, PDCD1LG2, and TNFRSF9 being significantly upregulated in the high-TMEscore subtype of all OS cohorts (Figures 7(b) and 7(c)).

**Figure 8:**
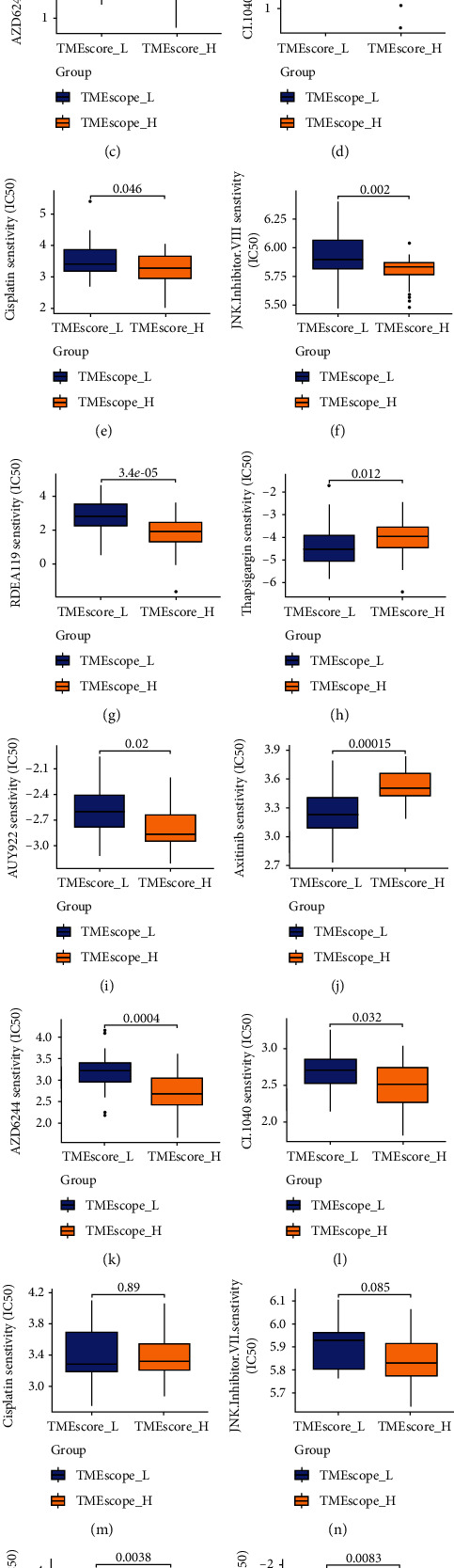
TMEscore is a prognostic biomarker and can predict the effect of chemotherapy drugs. (a–h) Results of drug sensitivity analysis in TCGA cohort. (i–p) Results of drug sensitivity analysis in the GEO cohort.

Next, we used pRRophetic to predict the IC50 of chemotherapies targeting MAPK signaling (axitinib, AZD6244, CI.1040, and RDEA119), JNK signaling (JNK inhibitor VIII), sarcoendoplasmic reticulum calcium ATPase pump (thapsigargin), and HSP90 (AUY922). This analysis revealed that except for axitinib and thapsigargin, these drugs were significantly more effective in high-TMEscore patients than in low-TMEscore patients. Similar observations were made upon validation analysis on dataset GSE21257. IC50 analysis showed that the low-TMEscore subgroup was less sensitive to cisplatin, while the high-TMEscore subgroup was more likely to benefit clinically (Figure 8).

### 3.7. Experimental Verification

**Figure 9:**
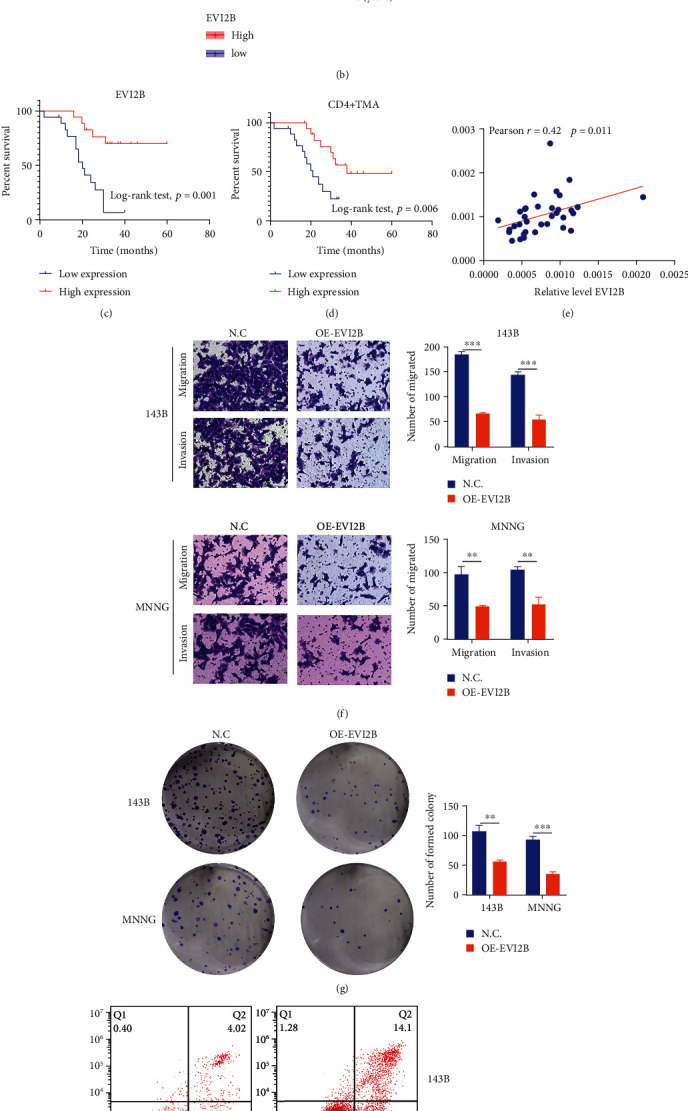
The role of EVI2B in OS. (a–c) Associations between EVI2B levels and survival outcomes in TCGA-OS, GSE21257, and Zhujiang cohorts. (d) KM survival curves for CD4 in the Zhujiang cohort. (e) Correlation between the expression of EVI2B and CD4 in the Zhujiang cohort. (f) The Transwell assay showed that EVI2B overexpression markedly suppressed osteosarcoma cell invasion and migration. (g) The plate colony formation assay was performed to investigate the effects of EVI2B on cell proliferation. (h) Effects of EVI2B on apoptosis were evaluated by flow cytometry. (i) Images of the subcutaneous tumor.

EVI2B, a characteristic gene of the TMEscore, is associated with prognostic outcomes for OS patients in TCGA and GSE21257 cohorts and verified in the Zhujiang cohort (Figures 9(a)–9(c)). CD4, as a landmark molecule to determine the expression of CD4+ TMA in vivo, was detected by RT-PCR in 36 OS samples from Zhujiang Hospital. We also found that that high expression of CD4 was remarkably associated with good prognosis of OS, and the higher the expression level of CD4, the better the prognosis (Figure 9(d)). We also examined the expression relationship between CD4 and EVI2B in the Zhujiang cohort and found a positive correlation between EVI2B and CD4 (Figure 9(e)). This suggests that with the high expression of EVI2B, there is more infiltration of CD4+ TMA. To further investigate the effects of EVI2B on OS cells, we performed in vitro and in vivo. Transwell assays revealed that OS cell migration and invasion were markedly suppressed in the OE-EVI2B group (Figure 9(f)). After the OE-EVI2B in 143B and MNNG cell lines, OS cell proliferations were significantly suppressed (Figure 9(g)), while flow cytometry showed that OE-EVI2B cell apoptosis levels were increased (Figure 9(h)). The overexpression experiment of EVI2B in nude mice also proved that high expression of EVI2B could antagonize the proliferation of OS (Figure 9(i)). Our laboratory data show that high expression of EVI2B is positively associated with increased CD4+TMA cell infiltration. Meanwhile, EVI2B and CD4+ TMA cells can also affect the pathogenesis of OS. On the one hand, the high expression of EVI2B can inhibit the malignant behavior of tumor proliferation and invasion. On the other hand, high expression of CD4+ TMA cells and EVI2B also improves the prognosis of patients with OS. These results confirm our previous relationship between EVI2B and CD4+ TMA cells and the effect of EVI2B as a component of TMEscore on OS. This proves that TMEscore can be used as a clinical indicator to guide the diagnosis and treatment of clinical patients.

## 4. Discussion

The TME plays essential roles in OS cell proliferation and progression [32]. The abundance of tumor-infiltrating lymphocytes in OS can predict patient responses to neoadjuvant chemotherapies and is correlated with OS prognosis [33]. However, there are studies that have reported opposing findings with regard to the effects of tumor-infiltrating lymphocytes on neoadjuvant chemotherapy responses or prognosis [34]. This opposing finding could be because tumor-infiltrating lymphocytes are heterogeneous populations [35, 36]. The TME heterogeneity affects various responses to clinical therapy; therefore, the TME is a potential biomarker for predicting clinical treatment effects and for screening tumor patients that can benefit from treatment, thereby informing clinical treatment.

We investigated the association between the KEGG pathway and survival outcomes and found that many immune-related pathways are associated with survival outcomes. Therefore, we evaluated the TME of OS. Two algorithms (ESTIMATE and ssGSEA) for evaluating TME further found that TME is closely related to tumor immune cell infiltration and patient survival in OS. This greatly attracted our exploration interest. Elevated lymphocyte infiltration levels, especially CD4+ T cells and CD8+ T cells, are associated with good prognostic outcomes after immunotherapy. In further analysis of the results, we found that high CD4+ TMA levels are associated with better survival outcomes. By comparing high- and low-TME groups, we found that CD4+ TMA was differentially expressed. Most of the cell types mentioned above have been reported in previous studies and play different roles in the development of tumor, such as immune evasion (myeloid-derived suppressor cells and Tregs) as well as regulation of tumor growth and invasion (CD8+ T cells and macrophages) in the TME. However, a limited number of studies on OS have investigated the roles of CD4+ TMA in oncogenesis.

We screened for potential signature genes by overlapping CD4+ TMA-related genes and DEGs and used LASSO analysis to identify key IRGs. Based on expression levels of C3AR1, EVI2B, FCGR2B, LILRA6, LPAR5, ITGAM, and WDR66, PCA analysis was performed on each sample to obtain TMEscore for every sample. To investigate the roles of TMEscore in OS patients, we used KM survival to establish differences in survival outcomes between high- and low-TMEscore groups. Comparable findings were obtained in the test set (GSE21257), implying that the TMEscore can be used to screen patients with poor prognostic outcomes.

Necchi et al. reported that elevated lymphocyte infiltration levels in cancer are associated with strong antitumor immune responses [37]. We found that in both experimental and validation groups, the TMEscore is associated with several antitumor cells, including macrophages, monocytes, and CD8+ T cells. To investigate differences between immune subtypes, we evaluated pathway activation and expressions of immune-related pathways among different groups and found that antitumor-related immune pathways were activated in the high-TMEscore subgroup. In conclusion, we postulate that the TMEscore, as a new biomarker, can predict prognostic outcomes based on the TME.

Then, we investigated the ability of the TMEscore to distinguish patient heterogeneity in order to personalize treatment.

Using TIDE's immune exclusion score, we found that the low-TMEscore subgroup had a greater chance of evading the immune system, implying worse clinical outcomes. By expanding TMEscore-related immune checkpoints, we were surprised to find that immune checkpoints were highly expressed in the high-TMEscore groups, suggesting that targeted immunotherapy can be used for personalized treatment.

Based on TMEscore grouping, various drugs that were effective in other tumor were not effective in OS, which was attributed to different drug sensitivities, implying that different drug interventions can be applied for personalized treatment.

To verify our findings, we investigated the effects of genes contained in the TMEscore, such as EVI2B, on OS. EVI2B regulates granulocyte function and differentiation by controlling cell cycle processes [38]. Many studies have shown that it can be used as part of the regulation of TME [39–41]. Experimentally, we found that EVI2B inhibits tumorigenesis and tumor development. In vivo analysis demonstrated that high expression of EVI2B can affect the growth of OS cells.

The above findings prove that the evaluated genes are associated with activities of immune cells, and they have the potential to influence the TME. However, the TME controls various biological processes. Therefore, it is important to evaluate the significance of multiple genes in the TME, to fully establish TME heterogeneity. This approach, which incorporates the major factors affecting the TME, can directly reflect differences in survival outcomes of OS patients and heterogeneities of responses to chemotherapy and immunotherapy.

## 5. Conclusions

In our study, we first demonstrated the important relationship between TME and OS patients, and then, we included the factors of TME and survival prognosis of OS as much as possible, obtained seven genes, and constructed TMEscore based on this. The OS subtype constructed by TMEscore can better distinguish the heterogeneity of OS and provide targeted therapy. In the experimental group (TCGA), we proved that TMEscore is a new biomarker, which can more accurately evaluate the prognosis of patients and clinical drug treatment, including immunotherapy. All the above results were confirmed in the validation group (GSE21257) and supported by laboratory data.

## Data Availability

All datasets are available from TCGA database (https://portal.gdc.cancer.gov/projects/TARGET-OS) and GEO database (https://www.ncbi.nlm.nih.gov/geo/query/acc.cgi?acc=GSE21257).
